# Advanced cervical dilatation as a predictor for low emergency cesarean delivery: a comparison between migrant and non-migrant Primiparae – secondary analysis in Berlin, Germany

**DOI:** 10.1186/s12884-018-2145-y

**Published:** 2019-01-03

**Authors:** Jürgen Breckenkamp, Eileen Marie Läcke, Wolfgang Henrich, Theda Borde, Silke Brenne, Matthias David, Oliver Razum

**Affiliations:** 1 School of Public Health, Department of Epidemiology & International Public Health, Bielefeld University, Bielefeld, Germany; 2 Obstetrics Clinics, Charité University Medicine Berlin, Campus Virchow-Klinikum and Mitte, Berlin, Germany; 3 Alice Salomon Hochschule Berlin, University of Applied Sciences, Berlin, Germany; 4 Clinic for Gynaecology, Charité University Medicine Berlin, Campus Virchow-Klinikum, Berlin, Germany; 5 Institute of General Medicine, Medical Faculty, Otto-von-Guericke-University Magdeburg, Magdeburg, Germany

**Keywords:** Maternal obesity, Gestational weight gain, Fetal macrosomia, Cesarean delivery, Immigrant women

## Abstract

**Background:**

Cesarean rates are higher in women admitted to labor ward during early stages rather than at later stages of labor. In a study in Germany, crude cesarean rates among Turkish and Lebanese immigrant women were low compared to non-immigrant women. We evaluated whether these immigrant women were admitted during later stages of labor, and if so, whether this explains their lower cesarean rates.

**Methods:**

We enrolled 1413 nulliparous women with vertex pregnancies, singleton birth, and 37+ week of gestation, excluding elective cesarean deliveries, in three Berlin obstetric hospitals. We applied binary logistic regression to adjust for social and obstetric factors; and standardized coefficients to rank predictors derived from the regression model.

**Results:**

At the time of admission to labor ward, a smaller proportion of Turkish migrant women was in the active phase of labor (cervical dilation: 4+ cm), compared to women of Lebanese origin and non-immigrant women. Rates of cesarean deliveries were lower in women of Turkish and Lebanese origin (15.8 and 13.9%) than in non-immigrant women (23.9%). In the logistic regression analysis, more advanced cervical dilatation was inversely associated with the outcome cesarean delivery (OR: 0.76, 95%CI: 0.70–0.82). In addition, higher maternal age (OR: 1.06, 95%CI: 1.04–1.09), application of oxytocic agents (OR: 0.55, 95%CI: 0.42–0.72), and obesity (OR: 2.25, 95%CI: 1.51–3.34) were associated with the outcome. Ranking of predictors indicate that cervical dilatation is the most relevant predictor derived from the regression model.

**Conclusions:**

Advanced cervical dilatation at the time of admission to labor ward does not explain lower emergency cesarean delivery rates in Turkish and Lebanese migrant women, despite the fact that this is the strongest among the predictors for emergency cesarean delivery identified in this study.

## Background

Rates of cesarean delivery worldwide are high and still rising. Depending on study region rates between 27.3 and 33.0% are reported [1–7]. Evidence shows that cesarean rates are higher in women admitted to the labor ward during the early stages of labor (i.e. with beginning cervical dilatation) than in those who were admitted at a later stage (i.e. with advanced cervical dilatation) [8–11], e.g. 10.3% vs. 4,2% [8] and 21.8% vs. 14.5% [11]. Moreover, Cheng et al. [12] found an increasing risk of cesarean delivery in association with the length of the first stage of labor; the longer the first stage, the higher the risk.

Cesarean rates are often higher in women who immigrated than in women born in the receiving country [13]. However, rates are not homogeneous in immigrant women of different origin. For example, women from sub-Saharan Africa are more likely to deliver by cesarean delivery than immigrant women from other low- and middle-income countries [14]. Disparities in cesarean birth rates between immigrant and non-immigrant women may occur because of non-medical factors such as communication barriers, less support and/or care practices during labor and delivery, female genital cutting, or cultural preference [15–17]. A different ethnicity or race thus is seen as a risk marker for cesarean delivery in women even if they have no obstetric risk factors [18, 19].

Findings regarding migrant status as risk factor for cesarean delivery are not consistent, however. David et al. [20] report that 1st generation immigrant women from Turkey and Lebanon delivering in Berlin/Germany had considerably lower crude rates of cesarean delivery (21.9 and 22.2%) than non-immigrant women (39.2%); the cesarean rates among 2nd and 3rd generation women were similar to those of non-immigrant women. To identify possible reasons for these unexpected findings we here assess 1) whether immigrant women from Turkey and Lebanon were admitted during later stages of labor (measured by advanced cervical dilatation) than non-immigrant women; and 2) whether later admission can explain lower cesarean rates in immigrant women – taking potential confounders (age, educational attainment, body weight, oxytocic agent) into account.

## Methods

### Setting, instruments, sample

We recruited women delivering in three secondary and tertiary care maternity hospitals of Berlin/Germany ((1) the Virchow Campus site of the Charité University Hospital, (2) the Vivantes Klinikum am Urban, and (3) the Vivantes Klinikum Neukölln) in a 12-month period 2011/12 (*n* = 8157). Minors (*n* = 105, 1.3%), tourists not resident in Germany (*n* = 24, 0.3%), women terminating a pregnancy, and women with miscarriages and stillbirths (fetal death in utero ascertained at hospital admission and before onset of labor, *n* = 106, 1.3%) were excluded. It was not possible to contact 363 women despite multiple attempts. Of the remaining 7559 women 381 declined to participate. We conducted face-to-face interviews and linked them with highly standardized obstetric process and outcome data from hospital databases. Linkage of available interview data with obstetric process and outcome data failed in 72 cases. Six women did not consent to the linkage of data sources. In total, 7100 women participated (response rate of 89.6%). This corresponded to 7334 birth data records because of twin and triplet births.

**Fig. 1:**
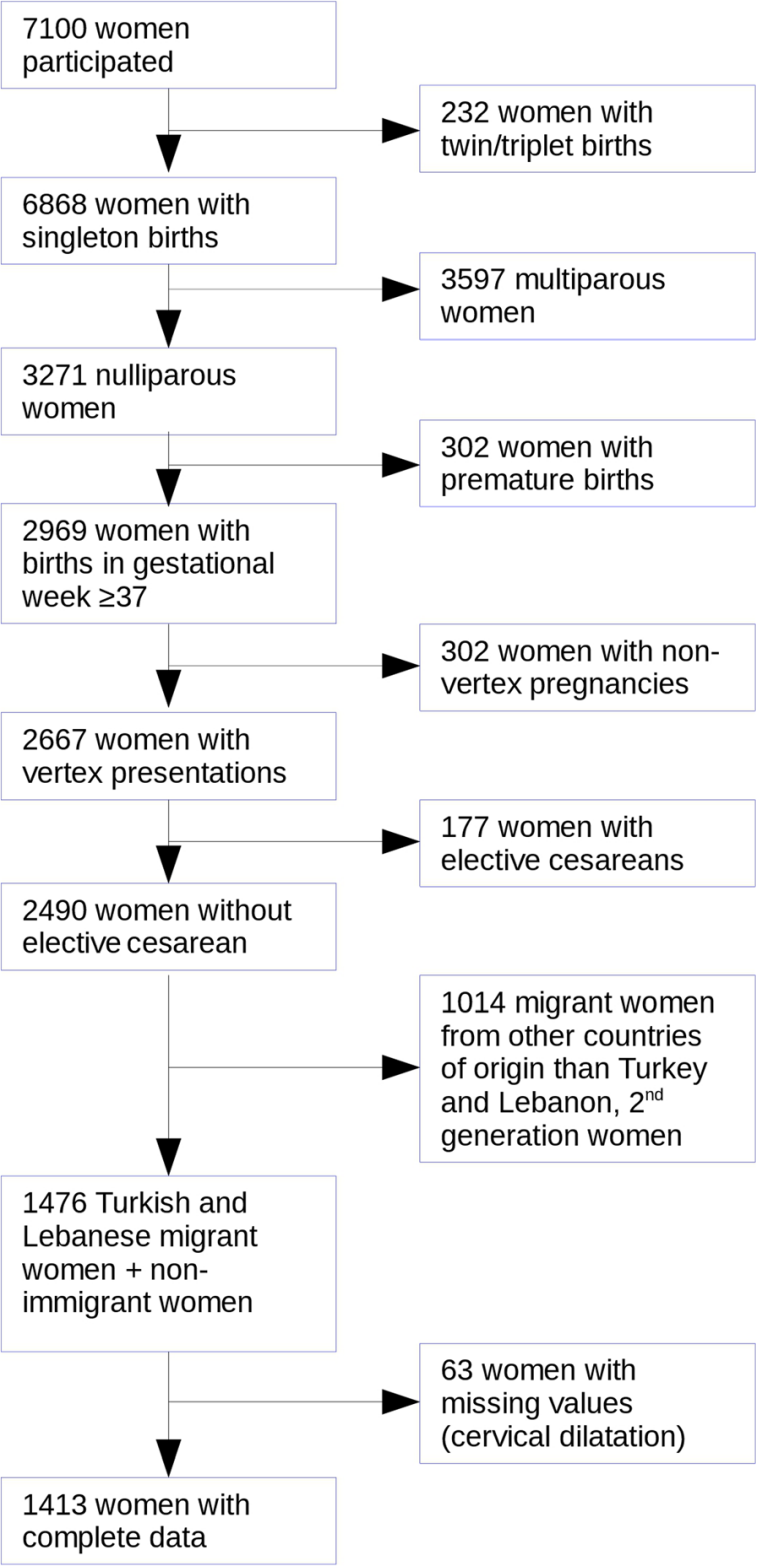
Flowchart of case recruitment, Berlin Perinatal study, 2011/12

For the analysis presented here, we considered only nulliparous women with vertex pregnancies and singleton birth, 37th week of gestation onwards. Women with elective cesarean delivery were excluded as they are not informative for our study question. We further restricted the sample to women with own migration experience (1st generation immigrants). Migrant women are a heterogeneous group. Health differences in this group might be larger than between migrant and non-migrant women. For this reason we selected women originating from Turkey and Lebanon (the two largest and only immigrant groups that allow separate analysis) and to women without a migration history (non-immigrant women). Also, 63 women without data on cervical dilatation were removed from analyses (see Fig. 1).

In the original study, interviews were conducted with each subject at two time points: on admission to the delivery room (T1) and on the second or third day postpartum in the maternity wards (T2). Questionnaires were available in German, Turkish, Kurdish, Arabic, and other languages. Translators were involved in case of language barriers. Nearly all women with migration background (193 of 205) were able to communicate with the obstetrician in German. For this analysis, only T1 data was used. Formally, the analyses reported here are secondary analyses as the original study question related to pregnancy outcomes such as frequeny of cesarean deliveries [20].

### Variables and statistical analysis

#### Cesarean delivery

The outcome variable was dichotomized, with “yes” comprising emergency cesarean delivery incl. crash cesarean; and “no” comprising normal vaginal delivery, forceps, and vacuum extraction.

#### Covariates

Covariates comprised known medical risk factors for the outcome. BMI (at admission) was coded as dummy variable: < 25 kg/m^2^, 25 - < 30 kg/m^2^, and ≥ 30 kg/m^2^. Medical treatment with an oxytocic agent was included as dichotomous variable. Age of pregnant women (continuous) and migration status (non-immigrant women, Turkish migrant women, Lebanese migrant women) were also included as covariates. Furthermore, we adjusted for confounding by socioeconomic factors by the best available proxy, educational attainment, measured by the highest graduation level. The variable was categorized into low (no education/primary school), medium (secondary education), and high attainment (technical collage/vocational school, a-level vocational diploma). Additionally, documented values of cervical dilatation (ranged from 0 cm to 10 cm) were used as continuous variable in the multivariate analysis.

#### Treatment of missing data

Among the final sample (*n* = 1413) information on maternal weight at the time of birth was missing in 8.6% (*n* = 122), and information on height in 11.0% (*n* = 156) of women. We assumed that missing data had occurred at random, i.e., that missings were not influenced by unobserved data. We applied imputation procedures using the average of five iterations with IVEware [21]. Imputation of maternal height was based on migration status and age; imputations of maternal weight were based on migration status, age, and height. Results of an analysis without imputed data do not differ substantially from the results given in Table 1 and Table 2 (see appendix, Table 4 and Table 5).

**Table 1:** Characteristics of the selected subsample of women, by migration status

	Turkish origin	Lebanese origin	Non-immigrants
*N*	133	72	1208
Age in years ^*** 1)^			
Median (range)	25 (18–45)	24 (18–41)	30 (18–44)
Highest educational level (%) ^*** 2)^			
No qualification/primary school	36 (27.1%)	14 (19.4%)	28 (2.3%)
Secondary school	72 (54.1%)	34 (47.2%)	530 (43.9%)
University / technical collage / vocational school / a-level vocational diploma	25 (18.8%)	24 (33.3%)	650 (53.8%)
Body Mass Index at admission (%) ^n.s. 2)^			
BMI < 25 kg/m^2^	17 (12.8%)	17 (23.6%)	235 (19.5%)
BMI < 30 kg/m^2^	66 (49.6%)	28 (38.9%)	546 (45.2%)
BMI ≥30 kg/m^2^	50 (37.6%)	27 (37.5%)	427 (35.4%)
Oxytocic agent ^n.s. 2)^			
Yes (%)	74 (55.6%)	33 (45.8%)	611 (50.6%)
Cervical dilatation ^* 2)^			
Median (range) in cm	2 (0–8)	2 (0–10)	2 (0–10)
Active phase of labor (≥4 cm) in %	21 (15.8%)	19 (26.4%)	315 (26.1%)
Delivery mode (%) ^** 2)^			
Normal vaginal delivery	75 (56.4%)	52 (72.2%)	685 (56.7%)
Vacuum extraction / forceps	37 (27.8%)	10 (13.9%)	234 (19.4%)
Emergency cesarean delivery	21 (15.8%)	10 (13.9%)	289 (23.9%)
Obstetric complications	*All participants (1403)*		
HELLP-Syndrome		6 (0.4%)	
Eclampsia		0	
Hemorrhage > 1000 ml		23 (1.6%)	
Sepsis		0	
Cardiovascular complications		0	
Uterine rupture		< 5 **	

* p < 0.05, ** *p* < 0.01, *** *p* < 0.001

1) Kruskal-Wallis test

2) Chi-square test

^**^ no detailed data due to data protection

**Table 2:** Indications for cesarean delivery

Rank	Turkish origin	*N* (%)	Lebanese origin	*N* (%)	Non-immigrant women	*N* (%)
1	Pathological CTG or auscultatory bad fetal heart tones	40 (30.1)	Pathological CTG or auscultatory bad fetal heart tones	17 (23.6)	Pathological CTG or auscultatory bad fetal heart tones	317 (26.8)
2	Protracted labor/obstructed labor in the expulsion stage	18 (13.5)	Green amniotic fluid	5 (6.9)	Protracted labor/obstructed labor in the expulsion stage	148 (12.3)
3	Protracted labor/obstructed labor in the dilation stage	9 (6.8)	Chorioamnionitis syndrome	4 (6.0)	Protracted labor/obstructed labor in the dilation stage	72 (6.6)
4	Chorioamnionitis syndrome	6 (4.5)	Protracted labor/obstructed labor in the dilation stage	4 (6.0)	Green amniotic fluid	43 (3.6)
5	Green amniotic fluid	3 (2.3)	Protracted labor/obstructed labor in the expulsion stage	4 (6.0)	Absolute or relative imbalance between child‘s head and mother‘s pelvis	29 (2.4)

Category “other birth risks” was not considered

#### Statistical analysis

Statistical distribution of cervical dilatation by migration status was plotted.

Multivariate logistic regression analysis was conducted to assess the association of cervical dilatation with the outcome “emergency cesarean delivery”. Covariates were educational attainment, BMI, oxytocic agent, maternal age, and migration status.

Linear regression analysis was performed for collinearity diagnostics. Effect measure modification was tested for with a logistic regression model. Neither collinearity nor effect measure modification were found.

We calculated standardized coefficients to rank the predictors (which had different scale levels) in the logistic regression analysis. The statistics software SAS 9.4 was used; the significance level was set at *p* < 0.05. Data protection regulations were observed in the survey and in the linkage to hospital data. The Charité Ethics Committee I, Berlin, approved the study (approval dated 18 February 2009; reference EA1/235/08).

## Results

Characteristics of the study population are provided in Table 1. There are noticeable differences in the distribution of age and educational level between the immigrant and non-immigrant women. Differences in obesity (BMI > =30 kg/m2) prevalence between migrant and non-immigrant women were small. 55.6% of Turkish migrant women, 45.8% of Lebanese migrant women, and 50.6% of non-immigrant women received oxytocic agents. At the time of admission to labor ward, a smaller proportion of Turkish migrant women (15.8%) were in the active phase of labor, compared to Lebanese (26.4%) and non-immigrant women (26.1%). Emergency cesarean delivery rates were lower in both immigrant groups (Turkish: 15.8%; Lebanese: 13.9%) compared to non-immigrant women (23.9%). Indications for an emergency cesarean delivery were among others failure to progress in labor, multiple birth, macrosomia combined with gestational diabetes, maternal diseases such as pre-eclamsia, s/p cesarean delivery, and anomalies of fetal presentation.

**Fig. 2:**
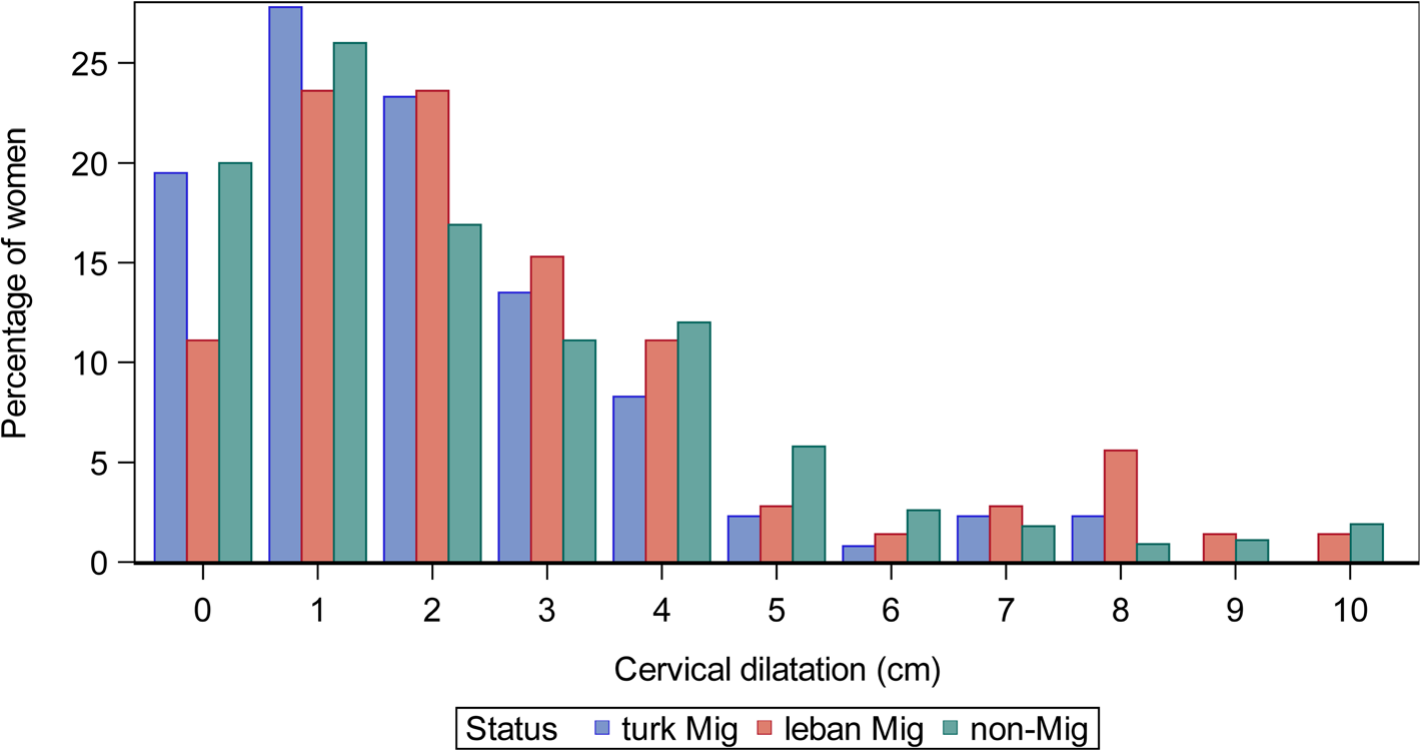
Cervical dilatation at admission, 1st generation Turkish and Lebanese women and non-immigrant women, Berlin Perinatal study, 2011/12

Figure 2 shows that cervical dilatation at admission most often is between 0 cm and 3 cm (before or in latency phase of labor). Overall, only small differences can be observed between immigrant and non-immigrant women, (Turkish origin vs. non-migrant women: chi-square test *p* < 0.01, Lebanese origin vs. non-migrant women: chi-square test *p* = 0.95), providing no evidence of a later admittance of 1st generation Lebanese migrant women but evidence for an earlier admittance of 1st generation Turkish migrant women (0 cm - 3 cm vs. 4 cm - 10 cm). When cervical dilatation was used as a continuous variable (0 cm - 10 cm), no statistically significant differences were found (Turkish origin vs. non-migrant women: Kruskal-Wallis test (median) *p* = 0.20; Lebanese vs. non-migrant women: Kruskal-Wallis test (median) *p* = 0.11). Women with premature rupture of the membranes are known for early admission. In our study 28.5% (335 of 1174) of women without premature rupture and 8.4% of women with a premature rupture admitted with > = 4 cm of cervical dilatation. A cesarean was performed in 23.1% of women without and 20.5% of women with premature rupture of membranes.

The most important obstetric complication is a hemorrhage (23 women). Pathological cardiotocography (CTG) or auscultatory bad fetal heart tones is ranked first of indications for cesarean (Table 2).

In the logistic regression analysis, advanced cervical dilatation (inverse association with an adjusted Odds Ratio (aOR) of 0.76, 95% CI: 0.70–0.82), maternal age (aOR: 1.06, 95% CI: 1.04–1.09), the application of oxytocic agents (inverse association with an aOR of 0.55, 95% CI: 0.42–0.72), and a BMI ≥30 (aOR: 2.25, 95% CI: 1.51–3.34) are statistically significantly associated with the outcome “emergency cesarean delivery” (Table 3).

**Table 3:** Odds ratios of emergency cesarean delivery among selected subsample of women, unadjusted and adjusted logistic regression model

Variable	OR	95% CI	*p*-Value	Standardized estimates
*Cervical dilatation (continuous)*	*0.76*	*0.70–0.82*	*< 0.0001*	0.342
*Maternal age (continuous)*	*1.06*	*1.04–1.09*	*< 0.0001*	0.198
1st generation Turkish migrants *	*0.63*	*0.37–1.08*	*0.0951*	0.075
1st generation Lebanese migrants *	*0.64*	*0.31–1.31*	*0.2229*	0.054
Oxytocic agent (yes) **	*0.55*	*0.42–0.72*	*< 0.0001*	0.198
BMI 25 to < 30 ***	*1.08*	*0.73–1.61*	*0.6911*	0.022
*BMI ≥ 30*	*2.25*	*1.51–3.34*	*< 0.0001*	0.214
Educational attainment (medium) ****	*0.78*	*0.40–1.54*	*0.4767*	0.068
*Educational attainment (high)*	*0.80*	*0.39–1.62*	*0.5333*	0.062

*References*: * = non-immigrant women, ** = oxytocic agent (no), *** = BMI < 25, **** = Educational attainment (low)

In addition, standardized estimates show that higher cervical dilatation is the most relevant predictor of an emergency cesarean delivery, followed by a BMI of 30 or above (Table 3).

Sensitivity analysis: Results of analysis with complete data show no substantial differences compared with the results of the imputed data set. This demonstrates that results are only slightly sensitive on changes in the study population.

## Discussion

At the time of admission to labor ward, a smaller proportion of Turkish migrant women was in the active phase of labor, compared to women of Lebanese origin and non-immigrant women. In the logistic regression analysis cervical dilatation was found to be the most relevant predictor of a cesarean delivery. Still, rates of cesarean deliveries were lower in women of Turkish and Lebanese origin than in non-immigrant women.

Early admission to delivery room before or in the latency phase (cervical dilatation of less than 4 cm) can lead to a mistaken diagnosis of failure to progress in labor [22]. This risk factor by now is well documented, but it continues to be observed in nulliparous women, even in the high-standard secondary and tertiary hospitals of Berlin/Germany participating in our study. Underlying may be expectations of observing a dilatation of 1 cm/hour, which are unreasonable for the actual, early stage of labor. This may lead to an overdiagnosis of dystocia and, subsequently, to an overuse of interventions aimed at accelerating labor progress – and ultimately to an emergency cesarean delivery [8, 10, 22, 23].

Cesarean deliveries are considered safe, but there are several risks associated with this procedure like infections, bleedings, allergic reactions to anesthetics, problems with the placenta in future deliveries or possible injuries to newborn.

A higher frequency of emergency cesarean deliveries among immigrants and ethnic minorities has been observed in several studies [24–27]; however, it tends to differ between sub-groups, according e.g. to country of origin or duration of stay [28]. A possible explanation could be an earlier admission of nulliparous immigrant women to labor ward, e.g. because of actual or expected communication problems. In our study, however, we did not find evidence for the hypothesis that 1st generation immigrant women would generally be admitted to labor ward at earlier stages of labor (as measured by cervical dilatation) than non-immigrant women. There is some evidence that this may be the case for Turkish, but not for Lebanese women: A larger proportion of Turkish nulliparous women is admitted to labor ward before the onset of the active phase, and the proportion of deliveries by vacuum extraction is twice as high as among Lebanese women. Nevertheless, crude cesarean delivery rates are considerably lower among women from Turkey and Lebanon compared to non-immigrant women. A lower chance of cesarean delivery remains visible in the logistic regression analysis but fails to reach statistical significance in our sample. Thus, the lower rates of cesarean delivery reported by David et al. [20] in the full data set of this study cannot be explained by later admission of 1st generation migrant women (at least not from Turkey and Lebanon) or by the immigrant status itself. Other factors not considered in this paper must therefore play a role, such as multiparity. The strengths of the study are the high participation rate (which makes selection bias unlikely) and the availability of detailed data from interviews and clinical documentation of perinatal variables. Sectio rates are lower than reported in the literature, which is attributable to the inclusion and exclusion criteria applied to this sample (see flowchart).

The main limitation is the sample size available for analysis due to exclusion criteria necessary to examine the study hypothesis. The cesarean delivery rates reported here are unlikely to be biased with regard to our study question. Exclusion criteria relate mainly to previous deliveries and different migration histories; the exclusion of women with elective cesarean deliveries is necessary because they are obviously not informative with regard to cervical dilatation. Moreover, given the heterogeneity of the immigrant population in Germany in terms of country of birth, migration background, and ethnicity, findings cannot be generalized to all immigrant women. Another limitation is that the sample is not representative for all German maternity hospitals; the way deliveries are organized may vary, thus incurring differing risks of emergency cesarean deliveries.

Turkish migrant women were more likely to have instrumental delivery compared to non-immigrant women, although cesarean delivery rates were certainly lower. This might suggest that other confounding factors help Turkish migrant women deliver instrumentally and may make the relationship between later admission and lower cesarean delivery rates in immigrant women obscure.

## Conclusion

Advanced cervical dilatation at the time of admission to labor ward does not explain lower emergency cesarean delivery rates in Turkish and Lebanese migrant women, despite the fact that this is the strongest among the predictors for emergency cesarean delivery considered in this study: With early admission, the chance of cesarean delivery increases, irrespective of migrant status. Obesity (BMI ≥30 kg/m^2^) and later maternal age follow in second and third place.

## Appendix

**Table 4:** Sensitivity analysis: Characteristics of the selected subsample of women, by migration status

	Turkish origin	Lebanese origin	Non-immigrants
N	109	53	1046
Age in years ^*** 1)^			
Median (range)	25 (19–45)	24 (19–40)	30 (18–44)
Highest educational level (%) ^*** 2)^			
No qualification/primary school	27 (24.8%)	10 (18.9%)	23 (2.2%)
Secondary school	64 (58.7%)	24 (45.3%)	446 (42.6%)
University / technical collage / vocational school / a-level vocational diploma	16 (16.5%)	19 (35.9%)	557 (55.2%)
Body Mass Index at admission (%) ^n.s. 2)^			
BMI < 25 kg/m^2^	14 (12.8%)	13 (25.5%)	198 (18.9%)
BMI < 30 kg/m^2^	53 (48.6%)	21 (39.6%)	484 (46.3%)
BMI ≥30 kg/m^2^	42 (38.5%)	19 (35.9%)	364 (34.8%)
Oxytocic agent ^n.s. 2)^			
Yes (%)	60 (55.1%)	24 (45.3%)	517 (49.4%)
Cervical dilatation ^* 2)^			
Median (range) in cm	1 (0–8)	2 (0–10)	2 (0–10)
Active phase of labor (≥4 cm) in %	17 (15.6%)	14 (26.4%)	275 (26.3%)
Delivery mode (%) ^* 2)^			
Normal vaginal delivery	60 (55.1%)	37 (69.8%)	591 (56.5%)
Vacuum extraction / forceps	30 (27.5%)	9 (17.0%)	198 (18.9%)
Emergency cesarean delivery	19 (17.4%)	7 (13.2%)	257 (23.9%)

* p < 0.05, ** p < 0.01, *** p < 0.001

1) Brown-Mood test

2) Chi-square test

**Table 5:** Sensitivity analysis: Odds ratios of emergency cesarean delivery among selected subsample of women, adjusted logistic regression model

Variable	OR	95% CI	p-Value	Standardized estimates
*Cervical dilatation (continuous)*	*0.78*	*0.72–0.84*	*< 0.0001*	0.305
*Maternal age (continuous)*	*1.06*	*1.03–1.09*	*< 0.0001*	0.195
*1st generation Turkish migrants **	*0.64*	*0.36–1.14*	*0.1305*	0.071
*1st generation Lebanese migrants **	*0.59*	*0.26–1.37*	*0.2224*	0.059
*Oxytocic agent (yes) ***	*0.61*	*0.46–0.81*	*< 0.0006*	0.137
*BMI 25 to < 30 ****	*1.04*	*0.68–1.57*	*0.8782*	0.009
*BMI ≥ 30*	*2.22*	*1.46–3.39*	*< 0.0002*	0.210
*Educational attainment (medium) *****	*0.66*	*0.32–1.35*	*0.2543*	0.115
*Educational attainment (high)*	*0.65*	*0.30–1.39*	*0.2671*	0.119

*References*: * = non-immigrant women, ** = oxytocic agent (no), *** = BMI < 25, **** = Educational attainment (low)

## Abbreviations

BMI: Body mass index
CI: Confidence interval
CTG: Cardiotocography
OR: Odds ratio
S/P: Status post
